# Diverse Activity of IL-17^+^ Cells in Chronic Skin and Mucosa Graft-Versus-Host Disease

**DOI:** 10.1007/s00005-019-00549-2

**Published:** 2019-06-08

**Authors:** Aleksandra Klimczak, Krzysztof Suchnicki, Mariola Sedzimirska, Andrzej Lange

**Affiliations:** 1 Hirszfeld Institute of Immunology and Experimental Therapy, Polish Academy of Sciences, R. Weigla 12, 53-114 Wroclaw, Poland; 2 Lower Silesian Center for Cellular Transplantation and National Bone Marrow Donor Registry, Wroclaw, Poland

**Keywords:** Graft-versus-host disease, Chronic GvHD, IL-17-producing cells, Skin and mucosa

## Abstract

Excessive inflammatory environment in a course of chronic graft-versus-host disease (cGvHD) is associated with T-cell trafficking into inflamed tissues. This study focused on the identification of IL-17-producing cells in the tissue biopsies of cGvHD patients. Forty-one biopsy specimens of cGvHD lesions of the skin (*n *= 27), gastrointestinal tract (*n *= 9) and oral mucosa (*n *= 5), examined in 24 patients, were morphologically defined according to the NIH criteria and analyzed for the presence of cellular infiltrations including: IL-17^+^, FOXP3^+^ and CCR6^+^ cells. IL-17^+^ cells were identified in 26/27 skin and in all gut and oral mucosa biopsies, being more frequent in mucosa lesions than in the skin (11/14 vs 14/26, respectively; NS: not significant). Double staining documented that CD138^+^/IL-17^+^ cells were commonly seen in the gut than in the skin (5/8 vs 3/11, respectively; NS). In the skin, cells expressing trafficking receptor CCR6^+^ were more frequent than IL-17^+^ cells compared to the mucosa (23/26 vs 2/13, respectively; *p *< 0.0001). CCR6 was present on a majority of IL-17^+^ cells in all examined skin biopsies but only in 6 out of 11 digestive tract biopsies (*p *= 0.0112). FOXP3^+^ cells were identified only in five patients (with mild lesions) at least in one biopsy. In this study group, results documented that local expansion of IL-17-producing cells in the digestive tract correlate with moderate and severe clinical symptoms of cGvHD, in contrast to the skin, where IL-17^+^ cells are rather scarcely present (*p *= 0.0301) and the course of cGvHD is slowly progressing with final organ deterioration.

## Introduction

Graft-versus-host disease (GvHD) is a life-threatening complication post-hematopoietic stem cell transplantation (HSCT) (Arora et al. 2016; Filipovich et al. 2005). This acute disease has a rather rapid course and even when responding well to the therapy may progress into a chronic disease. However, in some cases, chronic GvHD (cGvHD) is not preceded by acute disease. Chronic GvHD independently, whether it constitutes de novo manifestation of alloreactivity or follows acute GvHD (aGvHD), may have a diverse course from mild and limited to progressive and extensive disease. A progress of the disease in clinical staging is associated with deterioration in the immune system function. Therefore, cGvHD appears to be a syndrome of the immune system deregulation which is skewing the differentiation of the naïve CD4^+^ lymphocytes into effector cell activity at the expense of regulatory T cells (MacDonald et al. 2017; Sakaguchi et al. 2008). Inflammation, which leads to the destruction of affected tissues in a course of GvHD, is associated with pro-inflammatory cytokines production. It is known that interleukin (IL)-6 is produced at the site of inflammation in the GvHD process and its blood level is high and reflects severity of the disease (Karabon et al. 1995; Liu et al. 2010; Varelias et al. 2015). In the steady-state conditions, IL-6, if present in the milieu of differentiating lymphocytes, favors Th17 lineage. IL-17, secreted by Th17 lymphocytes, is a cytokine with pleiotropic functions important for host defense to bacterial and fungal infections especially in the gut (Geddes et al. 2011). It has been reported that IL-17 is the most potent inflammatory mediator in autoimmune-mediated tissue damage and in tissue injury after transplantation (Chung et al. 2012; Fujino et al. 2003; Gigante et al. 2011). In HSCT patients, IL-17 level increases in blood and at the tissue site involved in acute or cGvHD (Chen et al. 2007; Dander et al. 2009; Hill et al. 2010; Koh et al. 2016; Liu et al. 2013; Ratajczak et al. 2010). Our own study on IL-17 documented that Th17 cells are rather at a low level in the blood at the onset of aGvHD likely being marginalized in the affected tissues (Dlubek et al. 2010). To verify the hypothesis on the local accumulation of Th17 cells in the inflamed tissues, we focused on the identification of IL-17-producing cells in the tissue biopsies of cGvHD patients.

## Patients and Methods

Two hundred and twenty patients after allogeneic HSCTs (125 unrelated donors and 95 family matched donors) from our department were enrolled in this study. Clinical records from years 2004–2012 were collected retrospectively according to European Group for Blood and Marrow Transplantation guidelines by transplant physicians from our department.

Diagnosis and treatment of HSCT patients were standardized according to the National Institute of Health (NIH) criteria (Filipovich et al. 2005; Jagasia et al. 2015). In this retrospectively examined group, cGvHD developed in 81 patients. In 39 patients, cGvHD appeared as a de novo disease, but in 42 it was preceded by aGvHD. Forty-two cases had limited/mild and 39 extensive/severe cGvHD. Patients with mild/local disease were usually left untreated or if needed, topical treatment with or without low-dose oral cyclosporine A (CsA) and prednisone was installed.

The standard treatment procedure in extensive/severe disease involved three immunosuppressive drugs: CsA, mycophenolate mofetil (MMF) and prednisone. All patients on immunosuppression or having a low number of CD4^+^ cells (< 200/µl) received co-trimoxazol (alternatively pentamidine), imidazole and acyclovir prophylaxis.

Fifty-seven patients responded well to the therapy with at least stabilization of the local tissue involvement not progressing to the disability. Twenty-four patients (14 male and 10 female) had the progressive disease and the target organs were biopsied to assess the local inflammatory process activity. The clinical characteristics of this group of patients are summarized in Table 1. Among 24 patients, for whom biopsies were available, 15 received HSCTs from matched sibling (including one family haplotype matched) and 9 patients from unrelated donors (8 patients 10/10 matched at the allele level, 1 mismatched in one allele).

**Table 1:** Clinical characteristics of chronic GvHD patients

UPN	Age	Sex	Primary diagnosis	Donor (sex)	Conditioning regimen	T-cell depletion	aGvHD grade	cGvHD (day after HSCT)	GvHD prophylaxis^a^	Clinical symptoms of cGvHD	Clinical grade^b^	Outcome
494	55	M	AML	SIB (M)	RIC	ATG	0	De novo (730)	None	Skin, oral mucosa	Moderate	Died, +2508 days, relapse
590	48	M	NHL	SIB (F)	RIC	Campath	0	De novo 30 days after DLI	None	Skin, oral mucosa, eye	Severe	Died, +1660 days, cGvHD
640	41	M	CML	MUD (M)	MA	ATG	2	Continuation (100)	CsA, MMF, Steroids	Skin, oral mucosa, eye, lung	Severe	Died, +661 days lung cGvHD
672/2	59	M	AML	SIB (M)	RIC	–	2	Continuation (100)	Steroids	Skin, oral mucosa, eye	Moderate	Died, +937 days heart attack
677	40	M	CML	MUD (M)	RIC	ATG	1	Continuation (100)	CsA	Skin, oral mucosa	Mild	Alive, +1535 days
716	27	F	CML	SIB (M)	RIC	ATG	3	Continuation (100)	CsA	Skin, gut	Severe	Died, +138 days, cGvHD
764	46	M	ALL	SIB (F)	MA	–	2	Continuation (100)	CsA, MMF	Skin, eye, oral mucosa, gut	Moderate	Alive, 884 days
778	60	F	AML	SIB (F)	RIC	ATG	0	De novo (240)	None	Skin, oral mucosa	Mild	Alive, +768 days
779	46	F	AML	MUD (F)	MA	ATG	3	Continuation (100)	CsA, MMF, Steroids	Skin, oral mucosa	Mild	Died, +513 days, relapse
788	61	F	AML	SIB (M)	RIC	ATG	0	De novo (300)	CsA	Skin, oral mucosa, eye	Moderate	Alive, +632 days
794	20	M	MDS	MUD (M)	MA	ATG	2	Continuation (100)	None	Skin, lung	Severe	Alive, +521 days
819	53	M	AML	MUD (M)	MA	ATG	2	Continuation (100)	CsA	Skin, gut, oral mucosa	Moderate	Died, +330 days, cGvHD
733	40	F	MDS	SIB (F)	RIC	ATG	1	Continuation (140)	MMF, Steroids	Skin	Severe	Alive, +1405 days
671	54	M	AML	SIB (M)	RIC	Campath	0	De novo (776)	Steroids, CsA	Skin	Mild	Alive, +1924 days
723	45	M	MDS	MUD (F)	RIC	ATG	0	De novo (189)	CsA	Skin, oral mucosa	Moderate	Alive, +1471 days
682	52	F	CML	SIB (F)	RIC	ATG	0	De novo (447)	None	Skin, oral mucosa	Moderate	Alive, +1793 days
765	53	F	AML	SIB (F)	RIC	ATG	0	De novo (180)	MMF	skin	Mild	Died +1035 cGvHD
885	42	F	AML	MUD (F)	MA	ATG	3	Continuation (120)	Steroids, CsA, anti-TNF	Skin, gut, oral mucosa	Severe	Died +143 days, cGvHD, infection
508/1	46	M	CLL	SIB (M)	RIC	Campath	0	De novo (+1436)	CsA,	Skin,	Mild	Died +2413 days (sepsis)
852	49	M	AML	SIB (F)	MA	ATG	0	De novo (130)	CsA	Skin, oral mucosa	Moderate	Died +172 days, cGvHD, infection, sepsis
860	27	M	SAA	MUD (M) (9/10)	RIC	ATG	0	De novo (260)	CsA	Gut, liver	Severe	Died +406 days cGvHD, infection
878	54	F	AML	SIB (alt M)	RIC	ATG	1	Continuation (100)	Steroids, CsA	skin	Moderate	Died +325 days cGvHD, infection
869	43	F	AML	SIB (M)	RIC	ATG	2	Continuation (100)	Steroids	Skin, gut,	Moderate	Alive +539 days
896	30	M	AML	MUD (M)	MA	ATG	2	Continuation +(100)	None	Skin, gut, liver	Moderate	Died +208 days cGvHD

^a^GvHD prophylaxis at the time of biopsy collection

^b^Final clinical diagnosis was performed in accordance to NIH criteria (Filipovich et al. 2005)

*UPN* Unique patient number, *aGvHD* acute graft-versus-host disease, *cGvHD* chronic graft-versus-host disease, *AML* acute myeloblastic leukemia, *CML* chronic myeloblastic leukemia; *ALL* acute lymphoblastic leukemia, *MDS* myelodysplastic syndrome, *NHL* non-Hodgkin’s lymphoma, *CLL* chronic lymphocytic leukemia, *SAA* severe aplastic anemia, *SIB* sibling donor, *M* male, *F* female, *MUD* matched unrelated donor, *MA* myeloablative conditioning, *RIC* reduced intensity conditioning, *ATG* anti-thymoglobulin, *CsA* cyclosporine A, *MMF* mycophenolate mofetil

Twenty-two patients received peripheral blood progenitor cell mobilized by granulocyte colony-stimulating factor (G-CSF), whereas two patients unmanipulated bone marrow. The patients were on myeloablative conditioning that consisted of busulphan (16 mg/kg b.w. cumulative dose) and cyclophosphamide (120 mg/kg b.w. total dose) (Bu4 Cy2), or reduced intensity conditioning based on fludarabine (120 mg/m^2^ total dose) or melpharane (140 mg/m^2^). In all patients (except two), T cells were depleted by: ATG (2.5 mg/kg b.w.) to reach 0.01% of CD3^+^ blood level, or Campath (60 mg/kg b.w. cumulative dose). Acute GvHD prophylaxis included CsA (trough level 200 ng/L) with MMF (2000 g/day until + 30 day post-HSCT) if the transplant material had less than 1 × 10^6^/kg b.w. of CD34^+^ cells.

cGvHD was diagnosed from 79 to 1436 days post-HSCT (median: 270 days) and was assessed as mild (*n *= 6), moderate (*n *= 11) and severe (*n *= 7) grade. In 13 cases, cGvHD followed aGvHD, whereas in 11 patients, cGvHD appeared de novo. The clinical overview of this group of patients is summarized in the Table 2.

**Table 2:** Clinical overview of cGvHD patients

Characteristic	cGvHD (*N *= 24)
Median age of patient, years (range)	46 (20–60)
Sex of patient M/F	14/10
Sex of donor M/F	14/10
Donor	
Sibling (well matched/partially matched)	15 (14/1 alternative)
Unrelated (well matched/partially matched)	9 (8/1)
Diagnosis: AML/CML/MDS/NHL/CLL/SAA/ALL	13/4/3/1/1/1/1
Conditioning regimen	
Myeloablative	8
Reduced intensity	16
T-cell depletion	
ATG/Campath/none	19/3/2
HSC source	
PBPC	22
BM	2
cGvHD	
De novo	11
Continuation, prior aGvHD (Grade: I/II/III)	13 (3/7/3)
Organ involvement, grade: (mild/moderate/severe)	(6/11/7)
Skin	5 (3/1/1)
Skin + oral mucosa	7 (3/4/0) p = 0.0137
Skin + oral mucosa + eye	3 (0/2/1)
Skin + oral mucosa + gut	2 (0/1/1)
Skin + gut	2 (0/1/1)
Skin + oral mucosa + eye + gut	1 (0/1/0)
Skin + oral mucosa + eye + lung	1 (0/0/1)
Skin + lung	1 (0/0/1)
Skin + gut +liver	1 (0/1/0)
Gut + liver	1 (0/0/1)
Median post-transplant day of cGvHD diagnosis (range)	270 (79–1436)

*aGvHD* Acute graft-versus-host disease, *cGvHD* chronic graft-versus-host disease, *AML* acute myeloblastic leukemia, *CML* chronic myeloblastic leukemia, *ALL* acute lymphoblastic leukemia, *MDS* myelodysplastic syndrome, *NHL* non-Hodgkin’s lymphoma, *CLL* chronic lymphocytic leukemia, *SAA* severe aplastic anemia, *M* male, *F* female, *PBPC* peripheral blood progenitor cell, *HSC* hematopoietic stem cells, *BM* bone marrow

At the time of biopsy, in 18 out of 24 patients, cGvHD developed in spite of immunosuppressive pharmacotherapy installed either as a continuation of aGvHD treatment or cGvHD prophylaxis; patients received CsA and/or MMF or methylprednisolone (Table 1) according to the best individual response to treatment. In six patients with clinical symptoms of cGvHD, biopsy was taken prior to return/initiation of immunosuppressive therapy.

### Specimens and Immunohistochemistry

Altogether 41 biopsies were taken from sites with apparent cGvHD lesions of the skin (*n *= 27), the gastrointestinal tract (*n *= 9) and the oral mucosa (*n *= 5) were fixed in 10% formalin and embedded in paraffin blocks. Hematoxylin and eosin (H&E) staining was performed to classify the degree of morphological lesions according to the criteria proposed by NIH (Shulman et al. 2006, 2015). Inflammatory infiltrates were graded as follows: (1) mild when scattered lymphocytes were seen, (2) moderate when 20–30 lymphocytes were present and (3) extensive when lymphocyte clustered.

For immunostaining, 5-µm-thick sections were deparaffinized in xylene and ethanol gradient and placed for 30 min in preheated to 95 °C Target Retrieval Solution (Dako, Glostrup, Denmark): citrate buffer pH = 6.0 or Tris/EDTA buffer pH = 9.0 according to the recommended procedure.

Monoclonal antibodies (MoAb): CD3 (clone F7.2.38), CD4 (clone 4B12), CD8 (clone C8/144B), CD138 (clone Ml15), HLA-DR (clone TAL.1B5) (Dako, Glostrup, Denmark), FOXP3 (clone 236A/E7; Abcam, Cambridge, UK), CCR6 (clone 53103) and polyclonal antibody anti-IL-17 (goat IgG) (R&D Systems, Abingdon, UK) were applied. Tissue sections were incubated with the appropriate antibodies from 30 to 60 min. The binding of primary antibody for CCR6 and IL-17 was detected using DAKO LSAB-HRP System (Dako, Carpinteria, CA, USA), and for CD3, CD4, CD8, HLA-DR and FOXP3 using EnVision TM G/2System/AP (Dako, Carpinteria, CA, USA) in accordance with the manufacturer’s instructions. Slides were counterstained in hematoxylin and mounted in Faramount medium.

#### Two Color Staining

For double immunostaining, two methods, immunoenzymatic and immunofluorescence were used. Paraffin sections were deparaffinized as described above. For the presence of IL-17^+^/CD138^+^ cells, MoAb mouse anti-human CD138 and polyclonal antibody anti-IL-17 (goat IgG) and EnVision^TM^ G/2 Doublestain System (DAB^+^/Permanent Red) kit (Dako, Glostrup, Denmark) were used according to the manufacturer’s recommendation. IL-17^+^ cells were revealed using DAB chromogen, CD138^+^ cells were visualized using Permanent Red substrate, counterstained in hematoxylin and mounted in Faramount medium.

For immunofluorescence staining, the tissue sections were incubated with anti-IL-17 polyclonal antibody for 1 h, washed in PBS, and then rabbit anti-goat Ig/FITC (Dako, Glostrup, Denmark) was applied for 30 min. Next, the slides were incubated with MoAb mouse anti-human cytokeratin (clone MNF 116; Dako, Glostrup, Denmark) or CCR6 for 30 min, and the binding of antibodies was revealed by anti-mouse Ig/TRITC (Dako, Glostrup, Denmark) for 30 min. Slides were mounted in fluorescent mounting medium supplemented with 4′,6-diamidino-2-phenylindole for DNA-specific counterstaining.

Cellular infiltrates for: IL-17^+^, CD138^+^, and double positive IL-17^+^/CD138^+^, IL-17^+^/CCR6^+^ cells were assessed semi-quantitatively as the total number of cells in five high power fields (HPF) × 400 as follows: 0 lack of positive cells; + presented occasionally up to five cells in five HPF × 400; ++ 6–10 cells in five HPF; +++ more than 10 cells in five HPF.

Images were analyzed and recorded using immunofluorescence Olympus BX41 microscope, equipped with XC50camera and CellB software (Olympus, Japan).

### Statistics

Significances of differences of results were calculated using Fisher’s exact test and Statistica StatSoft Version 6.1 program. Differences between groups were considered significant at *p *< 0.05.

## Results

### Clinical Feature and Biopsy Diagnosis of cGvHD

The global assessment of cGvHD severity was based on the scoring system proposed by NIH criteria (Filipovich et al. 2005). Among 24 patients who experienced skin only or skin and oral mucosa lesions in six patients, cGvHD was diagnosed as mild, in five as moderate and in one as severe. The clinical severity increased when in addition to the skin and/or oral mucosa, other organs or tissues were involved such as gut, eye, lung and liver. In all these patients, moderate (*n *= 6) or severe (*n *= 6) clinical manifestations was diagnosed (*p *= 0.0137) see Table 2.

In all examined 27 skin biopsies, epidermal thickening, vacuolization of basal layer epidermal cells and apoptotic cells were microscopically evident. Lymphocytic infiltration were mild or moderate in 16 out of 27 skin biopsies (lymphocytes clustered close to dermal adnexa and dermal–epidermal junction, on average clusters of 5–30 lymphocytes), whereas in 11 out of 27 skin specimens, lymphocyte infiltrates were more extensive and associated with erythematous/violaceous flat-topped papules lesions (lichen planus-like eruptions). In all oral mucosa, biopsies (*n *= 5) apoptotic cells were also seen but lymphocyte infiltrations were much more prominent than in the skin and epithelial ulceration were noticed.

Examination of digestive tract biopsies (*n *= 9) revealed severe edema and destruction of glands. Lymphocytes were scattered throughout interstitial tissue, and colonizing the glands and/or ducts. Notably, in the skin as well as in the mucosa biopsies, cells with lymphoplasmacytic morphology were occasionally seen.

### Immunohistochemistry

#### Profile of Mononuclear Cells Infiltrations

**Fig. 1:**
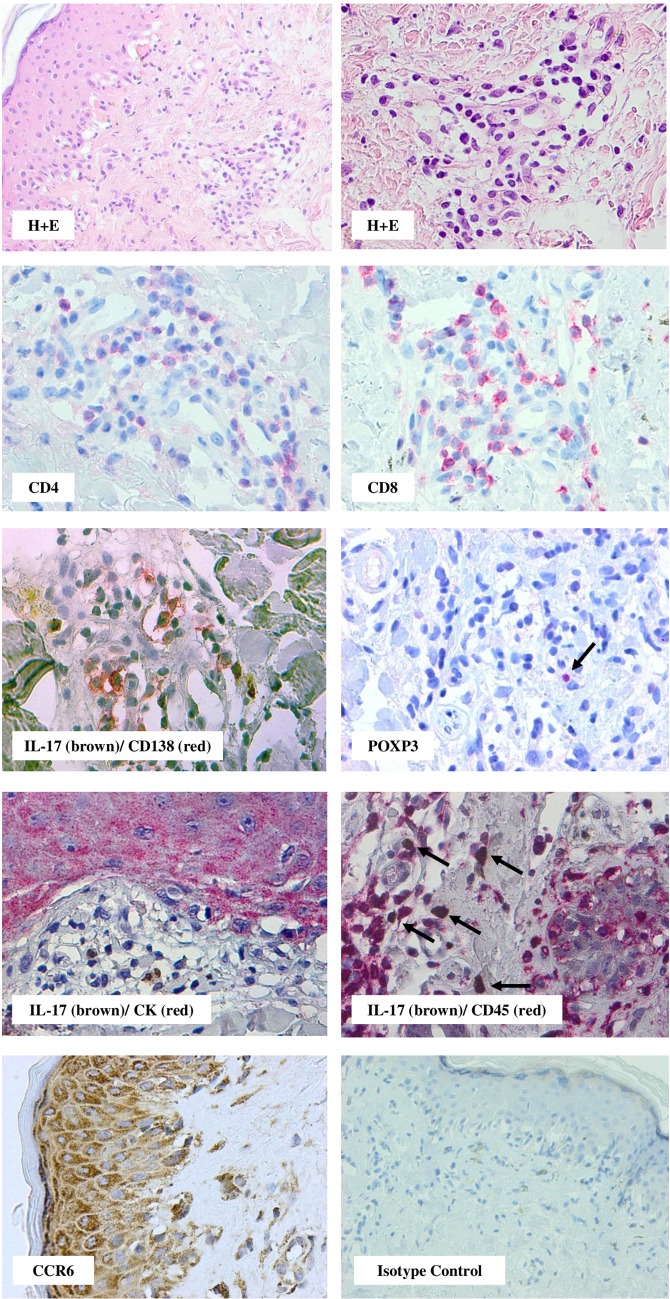
Skin biopsy specimen taken on day 361 after HSCT from the patient with mild lichen planus-like eruptions. Infiltrating cells were predominantly of T lymphocytes with similar proportions of CD4^+^ and CD8^+^ lymphocytes. Double immunostaining revealed the presence of IL-17-producing cells negative for CD45 and CD138 and negative for cytokeratin staining. Note the presence of single FOXP3^+^ cells (arrow). Epidermal keratinocytes, epithelium of eccrine coils and tissue infiltrating cells are CCR6 positive (red staining with Permanent Red, brown staining with diaminobenzidine-tetrahydrochloride (DAB); double stain with DAB/Permanent Red, magnifications: × 400, except H + E staining upper left and isotype control lower right staining: × 200)

**Fig. 2:**
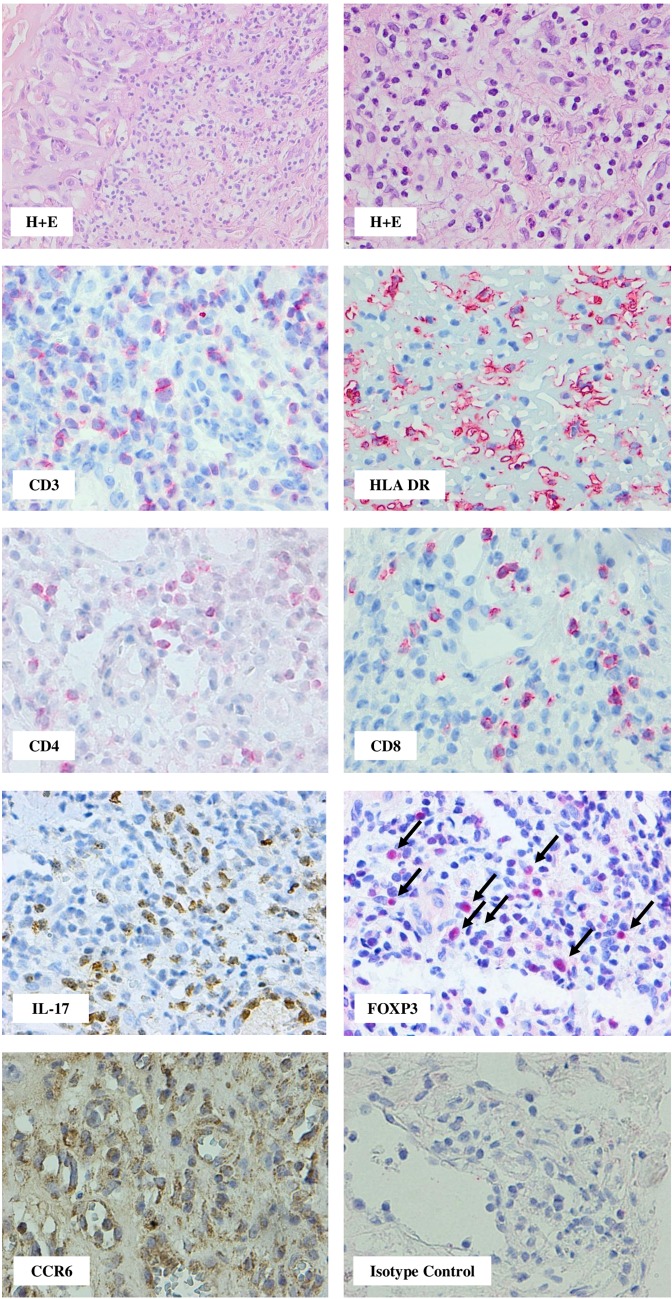
Oral mucosa specimen taken on day 361 after HSCT from patient with a mild course of cGvHD. CD8^+^ predominates over CD4^+^ cells. HLA-DR strongly expressed on a part of infiltrating cells and vessel endothelium. Please note the large numbers of both IL-17-producing cells and FoxP3^+^ cells (arrows). Epithelial cells of oral mucosa and a small proportion of infiltrating cells are weakly positive for CCR6 (red staining with Permanent Red, brown staining with diaminobenzidine-tetrahydrochloride (DAB); magnifications: × 400, except H + E staining upper left: × 200)

**Fig. 3:**
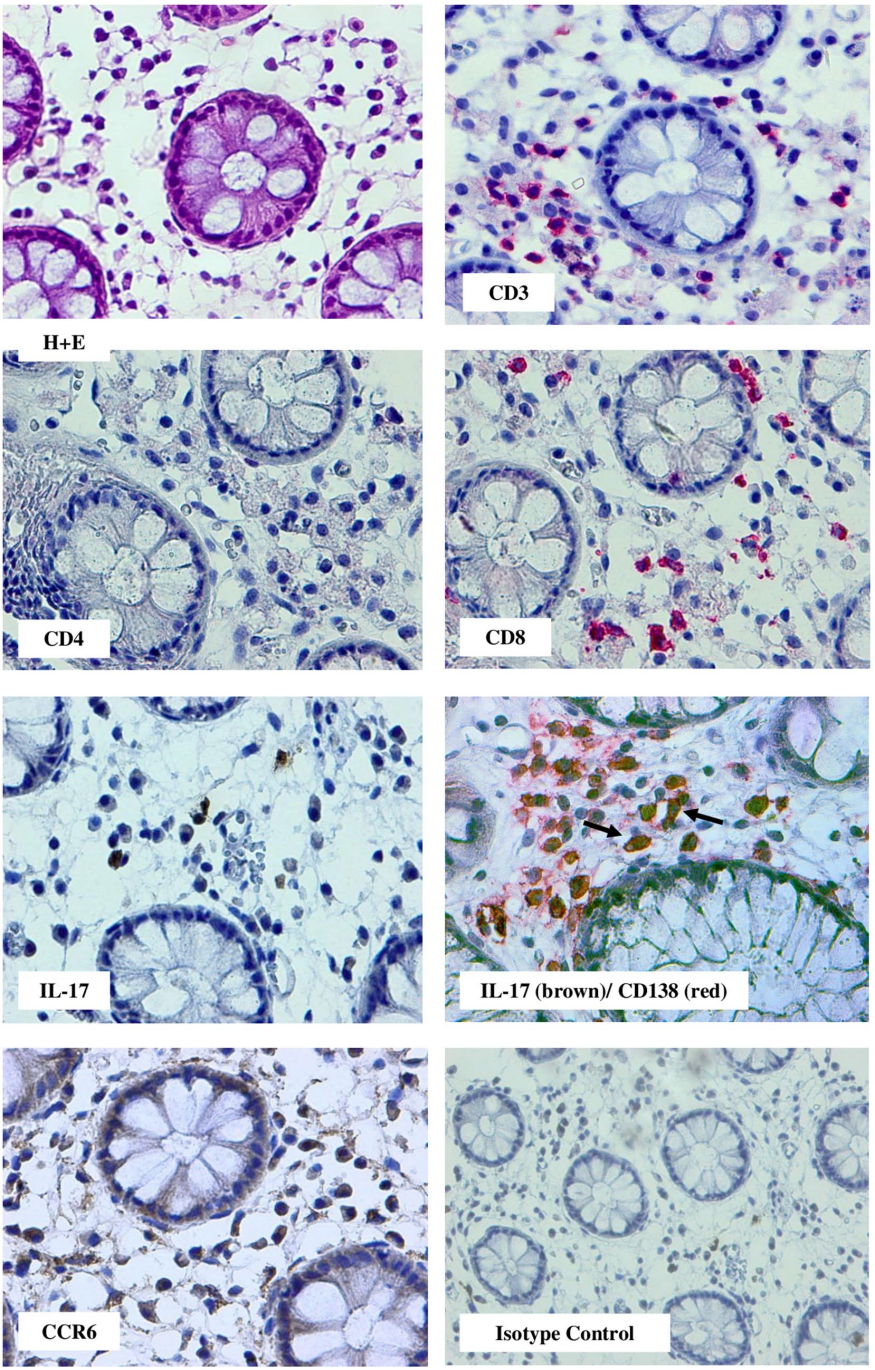
Rectum biopsy specimen obtained on day 141 post-HSCT from patient with moderate course of cGvHD having digestive tract mucosa lesions and diarrhea. Note, cell infiltration was composed of CD3^+^ cells and among them the CD8^+^ prevailed over CD4^+^. In the tissue infiltrates, IL-17-producing cells and cells expressing CCR6 were present. A proportion of cells were CD138^+^ and a double staining documented that some CD138^+^ cells of lymphoplasmacytoid morphology were also IL-17 positive (arrows); (red staining with Permanent Red, brown staining with diaminobenzidinie-tetrahydrochloride (DAB); double stain with DAB/Permanent Red, magnifications: × 400, except isotype control: × 200)

**Fig. 4:**
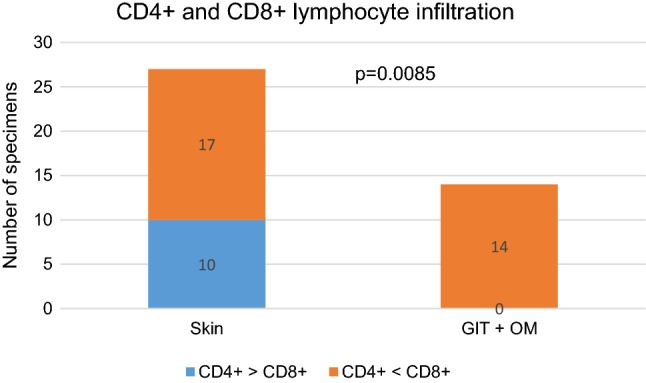
Cellular infiltrates in the tissues affected by cGvHD. Subpopulation of CD8^+^ cells predominate over CD4^+^ cells in all gastrointestinal (GIT) and oral mucosa (OM) biopsies compared to the skin in which CD4^+^ cells predominate in the proportion of specimens (*p *= 0.0085; Fisher’s exact test)

**Fig. 5:**
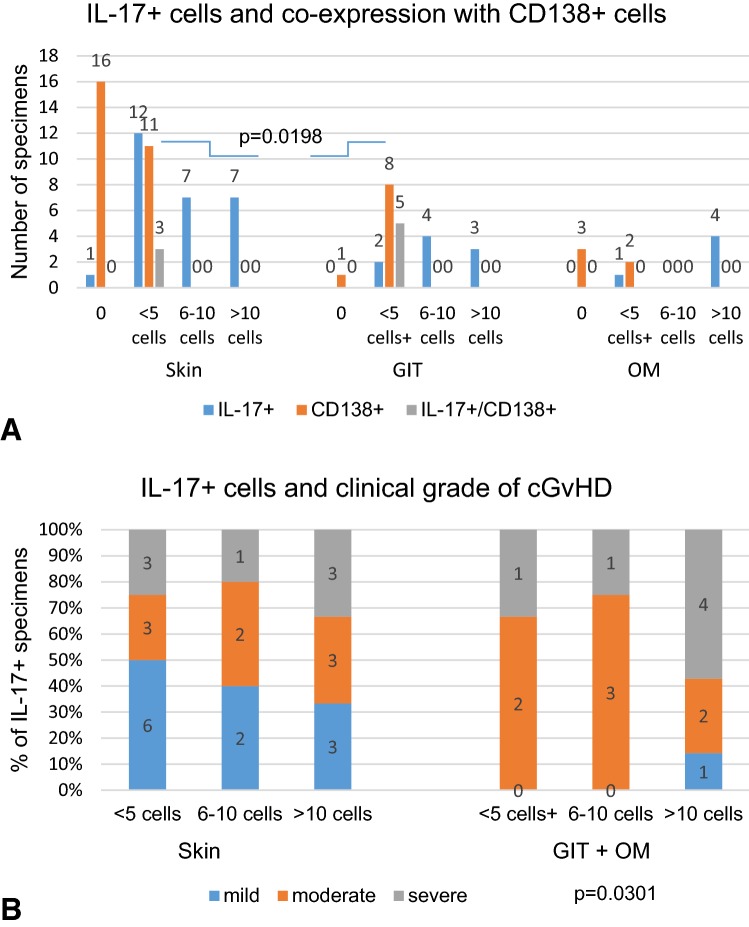
**a** IL-17^+^ cells were present in all gastrointestinal tract (GIT) and oral mucosa (OM) biopsies and in 26 skin specimens. A small proportion of cells with CD138^+^ phenotype were present more frequently in the GIT than in the skin and were IL-17^+^/CD138^+^ double positive. **b** The number of IL-17^+^ cells in the affected tissue is associated with a clinical grade of cGvHD. Mild stage of disease was diagnosed more frequently when skin lesions were observed, whereas moderate and severe symptoms of cGvHD were developed when GIT and OM were affected (*p *= 0.0301; Fisher’s exact test)

In the skin CD4^+^ prevailed over CD8^+^ lymphocytes in 10 out of 27 specimens, in contrast, in the oral mucosa and gut biopsies CD8^+^ predominated over CD4^+^ lymphocytes in all examined tissues (*p *= 0.0085) (Figs. 1, 2, 3, 4). Among mononuclear cells, a small proportion of cells of lymphoplasmacytoid morphology with CD138^+^ phenotype were present. These CD138^+^ cells were seen frequently in the gastrointestinal tract specimens (8/9 biopsies) than in the skin (11/27 biopsies; *p *= 0.0198) (Fig. 5A). In oral mucosa biopsies, CD138^+^ cells were detected in two specimens only as single scattered cells. A majority of lymphocytes infiltrating affected tissue were HLA-DR positive. Moreover, numerous small vessels in the vicinity of lymphocyte infiltrations, especially in the oral mucosa, revealed HLA-DR^+^ staining (Fig. 2).

#### IL-17-Producing Cells and FOXP3 Cells in Tissue Infiltrates

IL-17^+^ cells were seen in both gut and oral mucosa (14/14) and skin biopsies (26/27), but they were more frequent in the mucosa biopsies (Fig. 5a). The presence of an increased number of IL-17^+^ cells (according our grading a total of six or more cells in five HPF) was seen in 11 out of 14 gut and oral mucosa biopsies and in 14 out of 26 skin biopsies (NS: not significant) (Fig. 5a). In the oral mucosa, IL-17^+^ mononuclear cells were seen in the context of extensive lymphocytic infiltrates (Fig. 2). In the gut specimens, IL-17-producing cells were localized around the damaged crypts (Fig. 3).

The number of IL-17^+^ cells in the affected tissue was associated with the clinical grade of cGvHD. Skin lesions in the course of cGvHD was rather associated with mild stage of disease, whereas moderate and severe symptoms of disease developed when gastrointestinal tract and oral mucosa were affected (*p *= 0.0301) (Fig. 5b).

Double staining with anti-cytokeratin or CD45 and anti-IL-17 antibodies documented that IL-17 activity was exclusively associated with cellular infiltrates (Fig. 1). Double staining for CD138/IL-17 revealed that some of CD138^+^ cells were IL-17 positive and were commonly seen in the gut than in the skin (5/8 vs 3/11, respectively, NS; Fig. 1 and Fig. 3). Double positive CD138^+^/IL-17^+^ cells were not detectable in the oral mucosa biopsies.

In five patients, FOXP3 positive cells were present at least in one examined biopsy. Notably, none of these five patients had extensive lesions which were seen in 7 out of 19 patients lacking FOXP3^+^ cells at the tissue site.

#### IL-17-Producing Cells and Cells Expressing CCR6 Trafficking Receptor in the Tissue Infiltrates

**Fig. 6:**
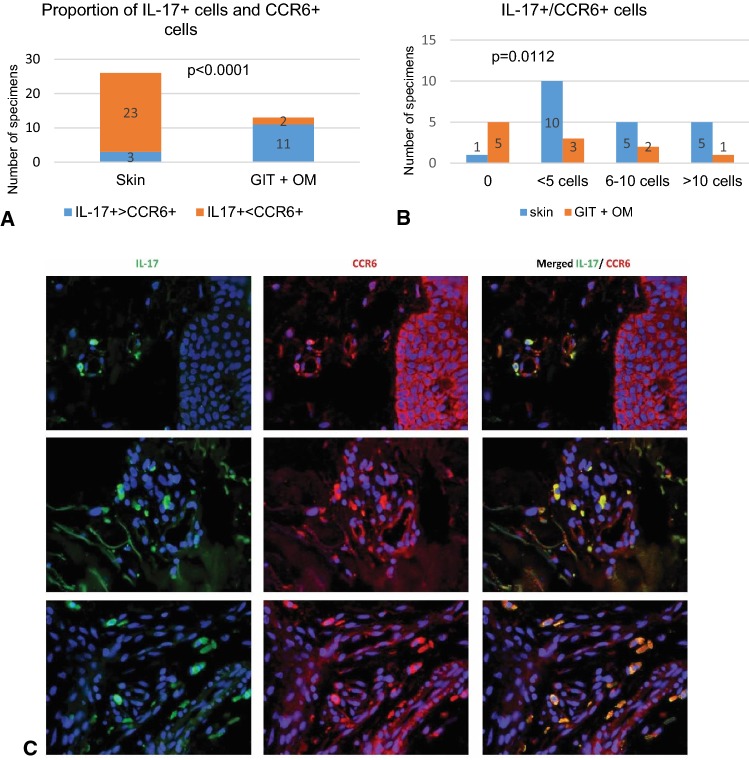
**a** Proportion of IL-17^+^ and CCR6^+^ mononuclear cells infiltrating the skin, gastrointestinal (GIT) and oral mucosa (OM). **b** Double immunostaining revealed that co-expression of CCR6^+^ with IL-17^+^ cells more frequently was seen in the skin compared to digestive tract biopsies (*p *= 0.0112; Fisher exact test). **c** Skin biopsy specimen obtained on day 1306 from patient with mild course of cGvHD revealed CCR6 positivity of numerous keratinocytes, eccrine coils epithelium as well as in tissue infiltrating cells. Many IL-17-producing cells expressed CCR6 (immunofluorescence double staining, green—FITC, red—Texas red, magnifications: × 400)

IL-17^+^ lymphocytes respond to the pro-inflammatory signals with the use of chemokine receptor CCR6. In all specimens, a proportion of mononuclear cells infiltrating the affected tissues were CCR6 positive. IL-17^+^ and CCR6^+^ cells, counted independently in the parallel sections in the skin, revealed more CCR6^+^ than IL-17^+^ cells (23/26 biopsies); in contrast, in the digestive tract IL-17^+^ cells prevailed over CCR6^+^ (11/13 biopsies; *p *< 0.0001) (Figs. 1, 6).

Double immunostaining (performed in 21 skin biopsies) showed that CCR6^+^ co-expressed with IL-17^+^ cells in 20 skin biopsies, but only in 6 out of 11 digestive tract biopsies (*p *= 0.0112) (Fig. 6).

## Discussion

According to the NIH criteria, acute and chronic GvHD are distinguishable rather by clinical and histological criteria but not according to the time elapsing after HSCT (Jagasia et al. 2015; Shulman et al. 2015). Severe clinical symptoms of cGvHD developed more often in patients who experienced prior aGvHD compared to these patients when the disease developed de novo. Most of the cGvHD patients examined in this study (92%) received G-CSF mobilized HSCT, which clinically has led to rapid hematopoietic reconstitution, but increased the risk of cGvHD as confirmed in clinical observations (Bensinger et al. 2001) and in experimental studies (Hill et al. 2010). The histopathology of cGvHD lesions seen in our examined group were concordant with prototype pictures introduced by NIH criteria (Shulman et al. 2006).

Previous reports, including own studies on the contribution of IL-17-producing cells in aGvHD, documented an increased number of IL17^+^ cells during immune reconstitution and decreased number of IL-17^+^ cells in the circulation at the time of clinical manifestation of aGvHD (Broady et al. 2010; Dlubek et al. 2010; van der Waart et al. 2012), and this suggest that they may have migrate to the affected tissues. Very few studies introduced tissue distribution of IL-17^+^ cells, in limited cases, in a course of cGvHD (Dander et al. 2009; Malard et al. 2014; Ratajczak et al. 2010) and these studies documented that Th17 cells may contribute to tissue damage. Detailed analysis of the present study documented that IL-17^+^-producing cells in a course of cGvHD plays a diverse role in different tissue compartments and were more frequent in the gut and oral mucosa than in the skin lesions. This would suggest that the prevalence of Th17^+^ cells in the digestive tract reflects a rather physiological representation of these cells in the gut as compared to the skin. Th17 cells are employed in both in physiological and pathological conditions. Physiologically, Th17 cells are seen along the mucosa barrier in the gut and they play an important role in the gut epithelial homeostasis and in the regulation of host immune response against a variety of pathogens (Asigbetse et al. 2010; Blaschitz and Raffatellu 2010). Studies on experimental model of germ-free or antibiotic-treated mice showed that in the absence of a luminal commensal microbiota, the number of Th-17 cells is significantly reduced (Atarashi and Honda 2011). Patients receiving conditioning regimen prior to transplantation are colonized by a number of bacterial species. In the view of this data, we can assume that the presence of IL-17^+^ cells at the site of cGvHD is due to the response to microorganisms rather than that to alloantigens. The commensal microbiota may have immunomodulatory influence on Th17 cells. Microbial products provide strong stimulation for local IL-6 production (Chung and Kasper 2010) which in the presence of transforming growth factor β facilitate differentiation of antigen-activated CD4 T cells into Th17 lymphocytes. Th17 immune response is associated with secretion of IL-17 and IL-22, neutrophils recruitment, and anti-microbial peptides induction in the early phase of inflammation, and constitutes a link between innate and adaptive immunity (Chung and Kasper 2010; Klimczak and Lange 2012). IL-17 is also considered as an innate cytokine produced by NK cells, γδ T cells, and CD8^+^ T lymphocytes (Geddes et al. 2011). Our studies also documented prevalence of CD8^+^ cells within the infiltrate-affected gut mucosa. However, it is also proposed that the expansion of Th17 cells into active phase of cGvHD is primarily due to a reduced frequency of Regulatory T (Treg) cells as confirmed in experimental model and in clinical observations (Chen et al. 2007; Dander et al. 2009; Rieger et al. 2006; Zorn et al. 2005). A defect of regulatory mechanism was also confirmed by the increased ratio of Th17/Treg in the human liver affected by cGvHD (Malard et al. 2014). Indeed, in the present study, the frequency of FOXP3^+^ cells in the affected tissues was low and did not follow the expansion of Th17-producing cells.

However, the situation is distinct in the lichen planus-like oral mucosa lesions. In contrast to cGvHD of the skin which is slowly deteriorating, in the oral mucosa, IL-17^+^ cells were often evident, and the intensity of infiltration of the IL-17^+^ lymphocytes was associated with severity of the clinical symptomatology. Patients with constant pain, hampering normal feeding, had a higher number of IL-17^+^ cells than those with milder clinical symptomatology of the oral lesions. Our observations documented that IL-17-producing cells participate in the active inflammatory process contributing to the tissue injury with painful symptomatology when oral mucosal lining is affected.

In contrast, the histological picture was more variable in the skin, beginning from scarce or denser papule eruptions to sclerodermic-like lesions. Active inflammatory process recognized as papullo-erythemic lesions is characterized by the presence of lymphocyte infiltrates and some of the cells were IL-17^+^, but they were much fewer in proportion than in the mucosa lesions. The profile of lymphocytes concomitant with Th17 also differs when we compare the skin and the gut lesions. In the skin, there are a large proportion of CD4^+^ lymphocytes, whereas in the gut, evident prevalence of CD8^+^ cells is visible. This observation may also add new insight to the understanding of the mechanism of cGvHD in different compartments, showing that in the skin and oral mucosa CD4^+^ lymphocytes contribute to the clinical symptoms by producing pro-inflammatory cytokines including (e.g., IL-17, interferon (IFN)-γ), but in the gut, cytotoxic attack on gut mucosa creates the clinical feature. It has been reported that after HSCT, a proportion of IL-17-producing T cells co-produce IFN-γ and these cells have the ability to differentiate into Th1 phenotype (Annunziato et al. 2007; Bahr et al. 2013; Gartlan et al. 2017). Therefore, IL-17-producing T cells, with or without IFN-γ-secreting properties, may contribute to GvHD without numerical expansion but via differentiation into Th1 phenotype (Bruggen et al. 2014; Dander et al. 2009; van der Waart et al. 2012).

According to the NIH criteria, one of the hallmarks of cGvHD is the presence of lymphoplasmacytoid cells (Shulman et al. 2006). In this study, we added novel information to the clinical role of these cells in a course of cGvHD. We proved that a proportion of CD138^+^ cells having lymphoplasmacytoid characteristics produce IL-17 and these were seen only in the gut. We believe that the presence of IL-17-producing cells in the gastrointestinal tract in cGvHD reflects the natural characteristic of the gut immune system which frequently employs these cells in the immune response. CD138^+^ cells were less frequent but still present in the skin lesions and they were IL-17 negative. Labeling for trafficking receptor CCR6 revealed that IL-17^+^ cells in the skin, but not in the gut, were CCR6 positive. Therefore, we can assume that CCL20 (the only ligand for CCR6) which is produced by macrophages involved in the local inflammatory process, is responsible for the attracting these IL-17^+^/CCR6^+^ cells to the inflammatory sites (Nakayama et al. 2001; van der Waart et al. 2012). This documented that CCR6 expression on the skin IL17^+^ cells plays a role as a homing receptor helping migration of IL-17-producing cells to the inflamed tissue (Singh et al. 2008; Wang et al. 2009).

Limitation of this study is the inadequate number of biopsies collected from oral mucosa or gut to compare the results with the same number of skin biopsies to identify immunopathological differences between different tissues affected by cGvHD. For this reason, this retrospective study, performed on a limited number of single-center patients, is considered as a pilot study and further studies need to be performed to assess the impact of IL-17-producing cells on tissue damage in the course of cGvHD. However, despite these limitations, the present study adds a novel observation for tissue-specific characteristics of cGvHD and shows that the physiological immune system has influence on the composition of lymphocyte infiltration in the affected organs. In the digestive tract, IL-17-producing cells are strongly represented and their presence is associated with clinical symptoms (pain, diarrhea) of cGvHD. In contrast, in the skin, IL-17^+^ cells are rather scarcely present and the course of cGvHD is slowly progressing with final organ deterioration.

## Abbreviations

CCR6: C–C motif chemokine receptor 6
CsA: Cyclosporine A
FOXP3: Forkhead box P3
GvHD: Graft-versus-host disease
cGvHD: Chronic graft-vs-host disease
HSCT: Hematopoietic stem cell transplantation
MMF: Mycophenolate mofetil
Treg: Regulatory T cells
